# Emerging Novel Biomarkers in Allergic Rhinitis: A Narrative Review

**DOI:** 10.7759/cureus.84705

**Published:** 2025-05-23

**Authors:** Tejaswi Mishra, KSBS Krishna Sasanka, Sree Sudha TY, Dheeraj Kumar, Suji PS, Gulistan Bano, Swaha Panda, Arnab Pal, Pradosh Kumar Sarangi

**Affiliations:** 1 Otorhinolaryngology-Head and Neck Surgery, All India Institute of Medical Sciences, Deoghar, Deoghar, IND; 2 Pharmacology, All India Institute of Medical Sciences, Deoghar, Deoghar, IND; 3 Radiodiagnosis, All India Institute of Medical Sciences, Deoghar, Deoghar, IND

**Keywords:** allergic rhinitis (ar), allergy immunotherapy, biomarkers, cluster of differentiation 39 (cd39), glucocorticoids, gzma, hypoxia-inducible factor (hif-1α), mirna-155, nasal inflammation, periostin

## Abstract

Allergic rhinitis (AR) is a highly prevalent, immunoglobulin E (IgE)-mediated inflammatory condition that significantly impacts global public health. While conventional biomarkers such as total and specific IgE and eosinophil counts are widely used, their limitations in diagnostic precision and treatment response prediction have prompted research into novel biomarkers. This review synthesizes emerging evidence from the past 15 years on innovative molecular markers implicated in AR pathogenesis and management. A comprehensive literature search identified preclinical and clinical studies investigating promising biomarkers, including periostin, microRNA-155, hypoxia-inducible factor 1-alpha (HIF-1α), Granzyme A (GZMA), CD39, and several serum and nasal fluid proteins such as orosomucoid (ORM), apolipoprotein H (APOH), and serpin family b member 3 (SERPINB3). Preclinical models highlighted their roles in immune regulation, barrier dysfunction, and inflammatory cascades. Clinical trials confirmed their diagnostic, prognostic, and therapeutic monitoring potential. Notably, periostin and miRNA-155 were associated with disease severity and responsiveness to sublingual immunotherapy, while proteins such as ORM and APOH predicted glucocorticoid (GC) treatment outcomes. CD39 and cytokine profiles showed promise for stratifying disease severity. Despite these advances, most biomarkers remain in investigational stages. Future work should emphasize multicenter validation and standardized protocols, and integration into biomarker panels for personalized AR care. This review underscores the evolving landscape of AR biomarker research and its translational potential in precision medicine.

## Introduction and background

Allergic rhinitis (AR), a prevalent immunoglobulin E (IgE)-mediated inflammatory condition affecting the nasal mucosa, arises from the immune system's overreaction to airborne allergens. This condition represents a significant global health concern, impacting an estimated 400 million individuals worldwide [1]. The incidence of AR has been on the rise, contributing to a substantial burden on individuals through diminished quality of life, sleep disturbances, reduced work and school productivity, and considerable economic costs. The interrelated nature of allergic conditions is evident in the frequent comorbidity of AR with asthma, observed in approximately 80% of asthmatic patients, highlighting the concept of a unified airway disease [2]. The significant prevalence and wide-ranging impact of AR underscore the critical need for improved diagnostic and management strategies, thereby emphasizing the relevance of research into novel biomarkers.

The pathogenesis of AR involves a complex interplay of immune responses. The initial sensitization phase occurs upon exposure to allergens, leading to the presentation of these allergens by dendritic cells to naive T helper (Th) cells. This interaction triggers the development of Th2 cells, which subsequently release cytokines such as interleukin (IL)-4 and IL-13. These cytokines promote the differentiation of B cells into plasma cells, resulting in the production of allergen-specific IgE antibodies [3]. Upon subsequent re-exposure to the same allergen, the effector phase is initiated. The allergen binds to the IgE antibodies already bound to high-affinity IgE receptors on the surface of mast cells and basophils. This cross-linking of receptors leads to the degranulation of these cells and the release of various preformed and newly synthesized inflammatory mediators, including histamine. Beyond mast cells and basophils, other immune cells, such as eosinophils, lymphocytes, and innate lymphoid cells (ILCs), along with a cascade of cytokines including IL-4, IL-5, and IL-13, contribute to the persistent allergic inflammation characteristic of AR [3]. A key aspect of AR pathophysiology is the compromised integrity of the nasal epithelial barrier, which facilitates the penetration of allergens into the nasal mucosa, perpetuating the inflammatory cycle [2]. A thorough understanding of this intricate pathophysiology is essential for the identification of relevant biomarkers that can accurately reflect the diverse facets of the disease process.

Currently, the clinical management of AR often relies on established biomarkers such as total and specific IgE levels, as well as eosinophil counts. However, these markers have limitations. For instance, the presence of IgE indicates sensitization to an allergen but does not always correlate with the manifestation of clinical allergy [4]. This highlights the need for the discovery and validation of novel biomarkers that can offer enhanced diagnostic accuracy, facilitate disease staging, enable more precise monitoring of treatment responses, particularly to allergen immunotherapy (AIT), and ultimately contribute to personalized medicine approaches in the management of AR. A significant unmet need in the field is the identification of reliable biomarkers that can predict which patients will respond favorably to AIT and determine the optimal duration of this treatment. The limitations of conventional biomarkers underscore the critical importance of discovering novel markers that can offer improved accuracy, predict treatment outcomes, and facilitate personalized approaches to managing AR.

## Review

Materials and methods

A comprehensive literature search was conducted using major databases such as PubMed, Scopus, Web of Science, and Google Scholar to carry out this review. The search strategy employed a combination of keywords such as "allergic rhinitis", "biomarkers", "novel biomarkers", "emerging biomarkers", "clinical trials", and "preclinical studies". These general terms were further combined with specific biomarker names identified from the initial review of the research material, including "periostin", "miRNA-155", "HIF-1alpha", "GZMA", "ORM", "APOH", "FGA", "CTSD", "SERPINB3", and "CC10". Boolean operators (AND, OR) were utilized to refine the search results and ensure the retrieval of the most relevant articles. A crucial aspect of the search strategy was the restriction of the publication timeframe to the period from 2010 onwards, thus taking into consideration the works done over the last 15 years. This systematic approach aimed to capture the recent advancements in the field of novel biomarkers in AR.

**Figure 1:**
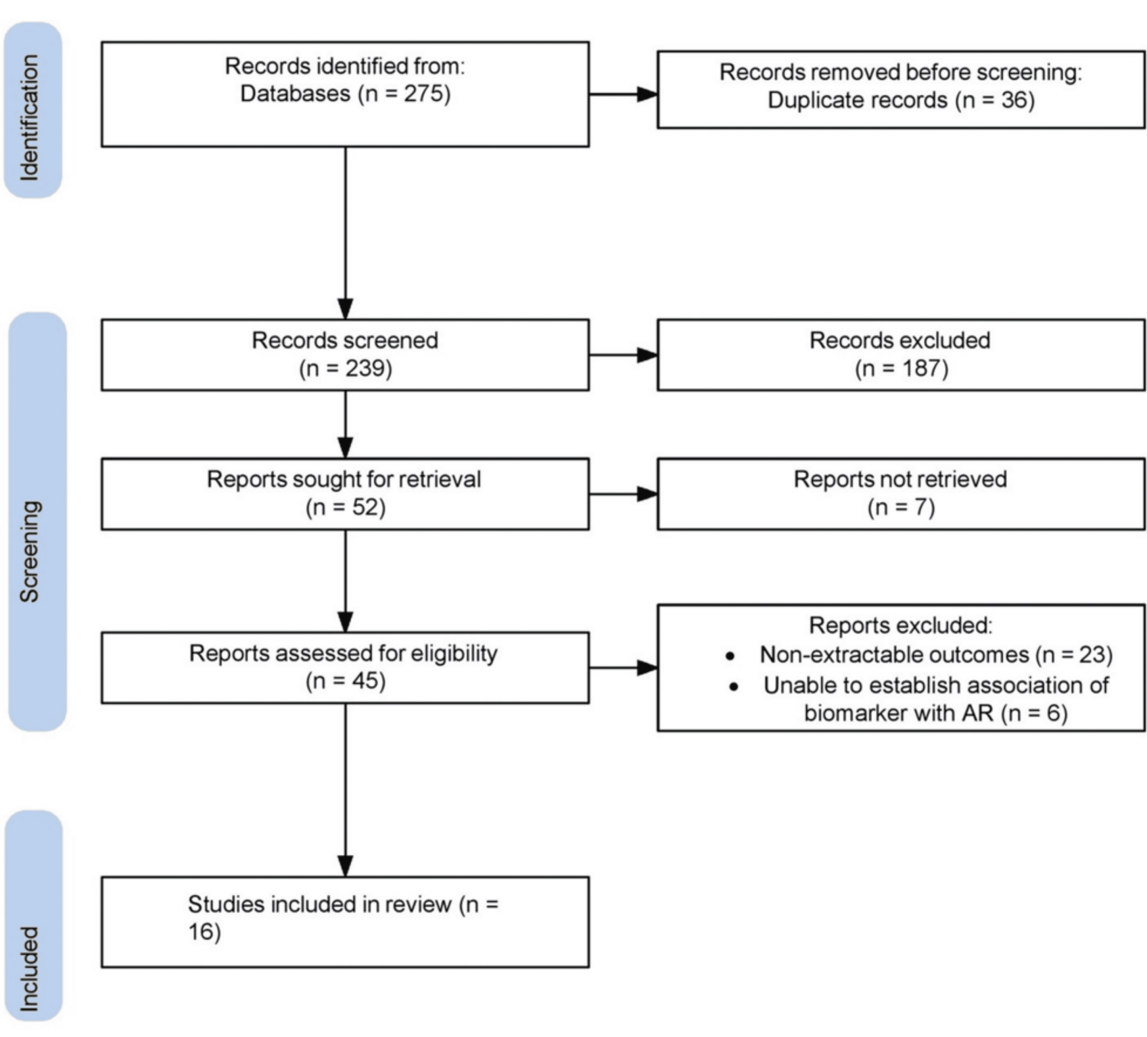
Flow diagram depicting study search and screening AR: allergic rhinitis

The selection of studies for inclusion in this review was focused on novel biomarkers in AR, extending beyond the well-established markers. Both preclinical studies, including in vitro research using cell lines and in vivo experiments with animal models, and clinical trials evaluating these novel biomarkers in human subjects with AR were considered. Additionally, review articles that provided significant insights into the topic were also included to offer a broader perspective. Studies that primarily investigated non-allergic rhinitis or focused solely on established biomarkers without exploring novel ones were excluded. Finally, only those studies with available full texts were included in the review. Figure 1 presents the flowchart outlining the study selection process.

The characteristics of the included studies are summarized in Table 1.

**Table 1:** Various biomarkers of AR studied over the last decade ORM: orosomucoid; APOH: apolipoprotein H; FGA: fibrinogen alpha chain; CTSD: cathepsin D; SERPINB3: serpin family b member 3; GC: glucocorticoids; HIF-1α: hypoxia-inducible factor-1alpha; VEGF: vascular endothelial growth factor; AR: allergic rhinitis; miRNA: microRNA; POSTN: periostin hub gene; CDC25A: cell division cycle 25A; PENK: proenkephalin; TNSS: total nasal symptom score; IL: interleukin; IFN-γ: interferon-gamma; TSLP: thymic stromal lymphopoietin; GZMA: granzyme A; FeNO: fractional exhaled nitric oxide; FnNO: fractional nasal nitric oxide

Authors	Year	Study design	Biomarker(s) investigated	Sampling method	Key findings
Wang et al. [5]	2011	Clinical	ORM, APOH, FGA, CTSD, SERPINB3	Nasal fluid protein	Identified these proteins as potential biomarkers for GC treatment response
Zhou et al. [6]	2012	Preclinical	HIF-1alpha, VEGF	Mice nasal mucosa	HIF-1α inhibition attenuated nasal allergic inflammation
Mo et al. [7]	2014	Preclinical & Clinical	HIF-1alpha, VEGF	Nasal mucosa, splenocytes	HIF-1α plays a role in AR in mice and humans; inhibition reduces allergic responses
Plank et al. [8]	2015	Preclinical	miRNA-155	Mice lungs	miR-155 is altered in the asthma model
Chen et al. [9]	2017	Preclinical	miRNA-155	Mice lungs	miR-155 silencing reduced AHR and airway inflammation in mice
Wang et al. [10]	2019	Preclinical	miRNA-155-5p	Epithelial cells, mouse model	miR-155-5p regulates allergic inflammation and epithelial barrier function
Pasca et al. [11]	2020	Review	miRNA-155	Plasma, nasal mucosa, epithelial cells	Reviews the role of miRNA-155 in inflammation
Hoshino et al. [12]	2021	Clinical	Periostin, IgE	Serum	High periostin levels are associated with an effective response to SLIT
Weidner et al. [13]	2021	Review	miRNAs including miRNA-155	Plasma, nasal mucosa, epithelial cells	Reviews the role of miRNAs in allergic diseases
Hao et al. [14]	2022	Clinical	POSTN, CDC25A, PENK	Nasal mucosa, blood	Identified these genes as potential biomarkers for AR diagnosis
Hammad et al. [15]	2021	Clinical	miRNA-155, IL-4	Serum	miR-155 levels were increased in AR children and correlated with TNSS
Selim et al. [16]	2022	Clinical	Periostin, IgE, sIL-2R, eotaxin	Serum	Periostin levels were increased in AR patients and changed after AIT
Jiang et al. [17]	2023	Clinical	CD39, IFN-γ, IL-5, IL-10, IL-13, IL-33,TSLP	Serum	Serum levels of CD39 and IL-10 showed potential for assessing the severity of the disease
Li et al. [18]	2025	Preclinical	GZMA	Mouse model, cell model	GZMA silencing reduced inflammation in AR models
You et al. [19]	2025	Preclinical	GZMA	Mouse model, cell lines	GZMA is a hub gene in AR and related to pyroptosis
Zhu et al. [20]	2025	Clinical	Periostin, IgE, FeNO, FnNO	Serum	Serum periostin was higher in AR patients and correlated with airway inflammation

Discussion

The quest for improved diagnostic and therapeutic strategies in AR has spurred significant research into biomarkers that extend beyond the traditional markers of IgE and eosinophils. Recent years have witnessed the emergence of various novel biomarkers, categorized broadly as matricellular proteins, microRNAs, hypoxia-related factors, and enzymes, among others. These markers hold the potential to refine our understanding of AR, enhance its management at different stages of the disease, and pave the way for more personalized interventions.

Preclinical Studies

Preclinical investigations, often conducted using in vitro cell culture models and in vivo animal models, primarily mice, have been instrumental in elucidating the potential roles of several novel biomarkers in the pathogenesis of AR.

Periostin

Periostin, a matricellular protein, has consistently shown elevated levels in preclinical models of allergic inflammation [21]. Studies have indicated its involvement in key processes such as eosinophil accumulation and the development of subepithelial fibrosis, both hallmarks of AR. Furthermore, periostin deficiency in mice has been shown to affect nasal tissue remodeling. These findings suggest that periostin plays a significant role in the inflammatory and structural changes associated with allergic airway diseases, positioning it as a potential therapeutic target [22].

Serpinb3a

Another notable study done in a mouse model of atopic dermatitis, allergen exposure led to increased expression of Serpinb3a, the mouse homolog of serpin family b member 3 (SERPINB3)/B4. It is a serine protease inhibitor, modulates the expression of the pro-inflammatory gene S100A8 in response to allergen exposure, thereby contributing to epithelial barrier dysfunction characterized by increased transepidermal water loss and inflammation. In Serpinb3a-deficient mice, IL-13-induced inflammation was significantly attenuated, highlighting Serpinb3a’s role in mediating barrier dysfunction, promoting early inflammatory responses in the pathogenesis of allergic disease [23].

MicroRNAs

MicroRNAs and small non-coding RNA (sncRNA) molecules that regulate gene expression have also garnered considerable attention. MicroRNAs (miRNAs/miRs) are a broad class of endogenous, non-coding RNAs that regulate gene expression at the post-transcriptional level by facilitating mRNA degradation or suppressing translation [11,13]. Dysregulation of miRNA expression has been associated with the pathogenesis of various human diseases. Among these, miRNA-155 has been found to be upregulated in preclinical models of allergic inflammation. Protein kinase inhibitor α (PKIα) has been identified as a direct target of miR-155-5p, exhibiting an inverse correlation with its expression. In a murine model of atopic dermatitis, miR-155-5p was markedly overexpressed in the skin, and its silencing resulted in a significant reduction in epidermal hyperplasia and inflammatory cell infiltration [10]. In the context of pulmonary function, miR-155-deficient mice displayed exacerbated airway remodeling. miR-155 has been shown to play a critical role in regulating allergic airway inflammation by modulating T-helper type 2 (Th2) responses via the transcription factor PU.1 and influencing the production of inflammatory cytokines, hallmark features of AR. Owing to its regulatory function in allergic inflammation, miR-155 has gained attention as a potential biomarker and therapeutic target in allergic diseases. Emerging evidence suggests that lentiviral vector-mediated delivery of siRNA targeting miR-155 may represent a novel and effective strategy for the treatment of allergic asthma [9].

Hypoxia-inducible Factor-1 Alpha

Hypoxia-inducible factor-1 alpha (HIF-1 alpha), a heterodimeric transcription factor activated under low oxygen conditions, has also been implicated in AR. HIF-1 alpha levels were found to be elevated in the nasal mucosa of mice during AR [6]. Notably, the inhibition of HIF-1 alpha in mouse models led to a reduction in nasal inflammation and the production of Th2 cytokines. Conversely, the stabilization of HIF-1 alpha exacerbated nasal allergic inflammation. These preclinical findings suggest that HIF-1 alpha plays a critical role in the inflammatory pathways of AR, making it a potential therapeutic target and a biomarker reflecting the disease's inflammatory status.

Granzyme A

Granzyme A (GZMA), a serine protease secreted by immune cells, has shown high expression levels in mouse models of AR. Western blot analysis confirmed the presence of GZMA in an AR mouse model [19,24]. In a cellular model of AR, silencing of GZMA resulted in the downregulation of pro-inflammatory cytokines IL-6, IL-4, and IL-5, accompanied by reduced apoptosis and enhanced proliferation of TNF-α-stimulated nasal mucosal epithelial cells. Conversely, GZMA overexpression produced opposing effects, promoting inflammatory cytokine production and apoptosis while inhibiting cellular proliferation [18]. These preclinical observations suggest that GZMA is involved in the inflammatory response associated with AR, warranting further investigation to determine its clinical relevance in human patients.

Glucocorticoid Treatment Response Proteins

Several proteins identified in nasal fluids have also shown potential as novel biomarkers in preclinical studies, particularly in relation to glucocorticoid (GC) treatment response. Orosomucoid (ORM), apolipoprotein H (APOH), fibrinogen alpha chain (FGA), cathepsin D (CTSD), and SERPINB3 have all been found to be differentially expressed in the nasal fluids of high and low responders to GC treatment [14]. Specifically, ORM, FGA, and APOH levels decreased significantly after GC treatment in high responders but not in low responders, suggesting their potential as predictive biomarkers for GC efficacy [5]. Apolipoprotein A-IV (apoA-IV), encoded by the APOH gene, was found to be increased in patients treated with sublingual immunotherapy (SLIT) and correlated with improved symptom and quality of life scores, as well as reducing histamine release from basophils in vitro [25]. SERPINB3 has been implicated in early inflammation and barrier dysfunction in preclinical models of atopic dermatitis and also showed differential expression in GC responders [5]. Clara cell secretory protein 10 (CC10) demonstrated reduced expression in AR models, while treatment with recombinant CC10 attenuated allergic airway inflammation in a mouse model of asthma, suggesting its potential as both a therapeutic and a biomarker [26,27].

Clinical Studies

The findings from preclinical studies have paved the way for clinical trials aimed at validating the potential of these novel biomarkers in human patients with AR.

Periostin

Periostin, an extracellular matrix (ECM) protein encoded by the POSTN gene and downstream of IL-4 and IL-13 signaling, has emerged as a key mediator in chronic allergic diseases. It plays a pivotal role in type 2 inflammation, contributing to fibrosis and airway remodeling associated with allergic inflammation. Periostin is now recognized as a reliable biomarker of type 2 inflammation and a potential predictor of airway eosinophilia. It exacerbates airway structural changes, enhances airway hyperresponsiveness, and shows considerable promise as both a diagnostic biomarker and a therapeutic target in asthma and AR. Notably, periostin-deficient mice with AR demonstrated reduced eosinophilic inflammation and subepithelial fibrosis, further underscoring its role in disease pathogenesis. Fractional exhaled nitric oxide (FeNO) is commonly utilized to assess eosinophilic inflammation in lower airway diseases and to identify patients with Th2-mediated airway inflammation, whereas fractional nasal nitric oxide (FnNO) serves as a marker of upper airway inflammation.

Studies have demonstrated that serum periostin levels are significantly elevated in patients with AR compared to healthy individuals, and these levels correlate with disease severity as well as with inflammatory markers such as FeNO and FnNO [20,28]. These observations suggest its potential in diagnosing AR and assessing its severity. Furthermore, high serum periostin levels have been associated with an effective response to sublingual immunotherapy (SLIT) in patients with AR, indicating its utility in predicting treatment outcomes [12]. As a potential biomarker for AR, serum periostin presents several clinical advantages, including ease of measurement, low inter-individual variability, and stable concentrations in adult populations. However, its utility in pediatric populations is limited by naturally elevated and age-dependent fluctuations in serum periostin levels, largely influenced by bone metabolism, which may confound interpretation in children [20].

MicroRNA-155

MicroRNA-155 has also been investigated in clinical trials. Serum levels of miR-155 were found to be increased in children with pollen-induced AR compared to healthy controls, and these levels showed a significant positive correlation with the total nasal symptom score (TNSS) [15]. These findings suggest that miR-155 could serve as a diagnostic biomarker for AR, particularly in pediatric populations, and that its expression may reflect the severity of nasal symptoms.

Glucocorticoid Treatment Response Proteins

The proteins ORM, APOH, FGA, CTSD, and SERPINB3, which showed promise in preclinical studies for predicting response to glucocorticoids, have also been evaluated in clinical trials involving patients with seasonal allergic rhinitis (SAR). Levels of these proteins in nasal fluids were found to differ significantly before and after treatment with glucocorticoids [5]. Specifically, ORM, FGA, and APOH demonstrated a significant decrease in high responders to GC treatment but not in low responders, further supporting their potential as biomarkers for predicting treatment response in SAR.

Peripheral Cytokines

Recent studies examining peripheral cytokine concentrations in patients with AR indicate that serum CD39 levels may be a strong indicator for diagnosing AR and assessing the severity of the condition. In comparison to the healthy control group, individuals with AR exhibited decreased levels of CD39 and interferon-γ, and increased levels of IL-13, IL-5, IL-33, and thymic stromal lymphopoietin(TSLP), as determined by multiplex cytokine analysis. The levels of serum CD39 and IL-10 demonstrated the potential in differentiating the varying degrees of disease severity [17].

While total IgE is an established biomarker for allergic diseases, clinical studies continue to explore its role in specific contexts. In children with chronic rhinitis, serum total IgE levels showed a statistically significant association with the severity of nasal eosinophilic inflammation, further supporting its relevance in assessing disease activity in this population [4].

Future Directions

Despite advances in identifying novel biomarkers, several key steps are needed to facilitate their integration into clinical practice. Large, multi-center trials are essential to validate current findings and ensure their applicability across diverse populations. Efforts should focus on standardizing measurement techniques and defining clinically relevant cut-off values to enhance diagnostic consistency. Exploring biomarker panels may improve diagnostic precision and treatment monitoring by reflecting various aspects of disease pathology. Additionally, prioritizing non-invasive sampling methods, especially in pediatric populations, could enhance patient compliance. The development of biologics targeting AR and the growing interest in seasonal proteomic profiling highlight new frontiers worth exploring. Finally, the role of biomarkers in guiding personalized therapies, including allergen immunotherapy and biologics, requires further investigation, particularly to address the ongoing need for predictors of treatment response.

## Conclusions

The landscape of AR biomarker research has expanded significantly in the last decade, with several studies exploring novel markers beyond traditional IgE and eosinophil counts. Preclinical investigations have highlighted the roles of matricellular proteins like periostin, regulatory molecules such as miRNA-155, hypoxia-related factors like HIF-1α, and enzymes like GZMA in the pathogenesis of AR. These studies have provided valuable insights into the underlying mechanisms of allergic inflammation and have identified potential therapeutic targets. Clinical trials have further validated the promise of some of these novel biomarkers. Serum periostin has shown potential as a diagnostic and severity marker for AR and as a predictor of response to sublingual immunotherapy. Similarly, circulating levels of miRNA-155 have been associated with AR diagnosis and symptom severity in children. Proteins identified in nasal fluids, including ORM, APOH, FGA, CTSD, and SERPINB3, have demonstrated potential in predicting the response to glucocorticoid treatment in patients with seasonal AR. Elevated levels of CD39 in the peripheral serum also demonstrated significant diagnostic potential for AR. Despite these advancements, incorporating these novel biomarkers into routine clinical practice requires further rigorous investigation. Future research should prioritize large-scale, multi-center clinical trials to validate the findings and establish the clinical utility of these markers.
